# Relationship of Computed Tomography Severity Score With Patient Characteristics and Survival in Hypoxemic COVID-19 Patients

**DOI:** 10.7759/cureus.22847

**Published:** 2022-03-04

**Authors:** Uday Yanamandra, Shivendra Shobhit, Debashish Paul, Bhavya Aggarwal, Praneet Kaur, Gayatri Duhan, Anurag Singh, Rajagopal Srinath, Puneet Saxena, Anil S Menon

**Affiliations:** 1 Internal Medicine, Armed Forces Medical College, Pune, IND; 2 Anesthesiology and Critical Care, Armed Forces Medical College, Pune, IND; 3 General Practice, Armed Forces Medical Services, New Delhi, IND; 4 Pulmonology, Army Hospital (Research And Referral; R&R), New Delhi, IND

**Keywords:** crp, neutrophil-lymphocyte ratio, d-dimer, ldh, inflammatory markers, ct severity score, computed tomography, covid-19

## Abstract

Background

Computed tomography (CT) scans and CT severity scores (CTSS) are widely used to assess the severity and prognosis in coronavirus disease 2019 (COVID-19). CTSS has performed well as a predictor in differentiating severe from non-severe cases. However, it is not known if CTSS performs similarly in hospitalized severe cases with hypoxia at admission.

Methods

We conducted a retrospective comparative study at a COVID-care center from Western India between 25th April and 31st May 2021, enrolling all consecutive severe COVID-19 patients with hypoxemia (peripheral oxygen saturation < 94%). Neutrophil-lymphocyte ratio (NLR), C-reactive protein (CRP), interleukin-6 (IL-6), lactate dehydrogenase (LDH), D-dimer, ferritin, and CT thorax were done within 24h of admission before being initiated on any anti-COVID-19 therapy. CTSS was calculated by visual assessment and categorized into three severity categories and was correlated with laboratory markers and overall survival (OS). Statistical analysis was done using John's Macintosh Project (JMP) 15.0.0 ver. 3.0.0 (Cary, North Carolina).

Results

The median age of the study population (n-298) was 59 years (24-95) with a male preponderance (61.41%, n=183). The 15 and 30-day survivals were 67.64% and 59.90%, respectively. CTSS did not correlate with age, gender, time from vaccination, symptoms, or comorbidities but had a significant though weak correlation with LDH (p=0.009), D-dimer (p=0.006), and NLR (p=0.019). Comparing demographic and laboratory aspects using CT severity categories, only NLR (p=0.0146) and D-dimer (p=0.0006) had significant differences. The 15d-OS of mild, moderate, and severe CT categories were 88.62%, 70.39%, and 52.62%, respectively, while 30d-OS of three categories were 59.08%, 63.96%, and 49.12%, respectively.

Conclusion

Among hospitalized severe COVID-19 patients with hypoxemia at admission, CT severity categories correlate well with outcomes but not inflammatory markers at admission.

## Introduction

Coronavirus disease 2019 (COVID-19), caused by the novel severe acute respiratory syndrome coronavirus 2 (SARS-CoV-2), has had a devastating impact on the global population in the last two years. A computed tomography scan of the thorax (CT thorax) in high-resolution CT protocol (HRCT) gives valuable information about the extent and stage of the disease and evaluates pre-existing/co-existing cardio-thoracic abnormalities that may affect the management plan and prognostication [1-3]. Since the start of the pandemic, there has been an ongoing debate on the indications, timing, and utility of CT thorax in COVID-19 with recommendations now favoring this imaging modality in moderate to severe disease [4]. It has previously been shown that the extent of changes seen on CT thorax correlates well with the severity of disease, outcomes, and inflammatory markers [5-10]. Patients with severe disease are likely to have higher CT severity scores (CTSS) than those with mild illnesses [11]. CTSS is better associated with hospital admission and intensive care unit (ICU) requirements than with mortality [9]. Among hospitalized patients, survivors have been found to have a lower CTSS compared to non-survivors [12]. However, 40% of subjects in the aforementioned study did not have hypoxia at admission. It is not clear whether CT thorax can be a prognostic marker among patients who present with severe disease with hypoxia. We conducted a retrospective study in a cohort of hospitalized severe COVID-19 cases to see the correlation of CT thorax with outcomes and laboratory parameters.

## Materials and methods

Study design

The study was a retrospective, observational cross-sectional study conducted at a COVID-care center from 25th April to 31st May 2021. This center catered for reverse transcriptase-polymerase chain reaction (RT-PCR)-positive severe-COVID cases. Severity was defined by pulse oximeter oxygen saturation (SpO2) level at room air of < 90%, or respiratory rate > 30 breaths per minute at initial presentation. Hypoxemia was defined as SpO2 < 94%. The institutional ethical committee approved the study, and informed consent was obtained for all the patients. The study was done in accordance with the Declaration of Helsinki.

Subjects

All consecutive patients admitted to this study center were screened for inclusion in the study. During the study period, there was a massive surge of COVID-19 cases and there was a shortage of ICU beds and trained staff. It was the institutional protocol to subject all severe COVID-19 cases to HRCT chest. This was done to assist in triaging, allocate ICU beds, and expedite evaluation for other co-existing lung conditions that needed attention. Thus, all severe cases were subjected to HRCT chest unless they were too unstable for undergoing a CT scan. All those patients who had severe disease and underwent HRCT scanning within 24h of admission and at least four days post-development of any of the COVID symptoms were included in the study. Patients who had already been started on glucocorticoids were not included for analysis. All initial chest HRCT scans were performed on the day of the patients' presentation using a volume computed tomography (VCT) GE 64 scanner (General Electric Company, Boston, Massachusetts). The supine position was adopted for scans with a single breath-hold. Contiguous image acquisition was carried out, and the images were subsequently reconstructed into a lung window in the sharp algorithm (60-80 kernel) and a soft tissue window (20-30 kernel) with a section thickness of 1.5 mm. Radiologists with more than eight years of experience studied the scans for qualitative and quantitative assessment in each patient. The scans were assessed for the presence or absence of typical findings of COVID-19 pneumonia as defined by the Radiological Society of North America (RSNA) consensus statement [1,13]. CTSS was then calculated using the visual assessment of each lobe involved [14-15]. Each lobe was evaluated for the proportion of lobe evaluated and scored as 0, 1, 2, 3, 4, or 5 depending on the involved proportion of 0%, <5%, 5-25%, 26-50%, 51 to 75%, and greater than 75%, respectively. The scores of each of the five lobes were totaled to calculate the total CT severity score [5-6,16]. These patients were further classified based on their CT severity score as mild (≤7), moderate (7-17), and severe cases (>17) [6]. Those patients who were unable to undergo CT scan for any medical contraindication, hemodynamic instability, or were unwilling to undergo HRCT Chest were excluded.

Methodology

A detailed history regarding comorbidities (hypertension, cardiovascular diseases, respiratory disorders, hypothyroidism, and central nervous system (CNS) disorders), COVID vaccination status, days from last vaccination, and COVID symptomatology (fever, dyspnea, cough, expectorations, hemoptysis, myalgia, headache, chest pain, ageusia, and diarrhea) were recorded at the time of admission along with the medical history of the patient. Neutrophil-lymphocyte ratio (NLR), C-reactive protein (CRP), interleukin-6 (IL-6), lactate dehydrogenase (LDH), D-dimer, and ferritin were measured at admission and within 24 hours of HRCT chest. All the patients were managed with currently prevailing protocol-based therapy guided by national guidelines, including steroids, low molecular weight heparin (LMWH), and vitamin and mineral supplements. Discharge criteria were uniformly applied as per the prevalent local guidelines. The outcome parameters included days of hospitalization and mortality.

Statistical analysis

The data were analyzed using John's Macintosh Project (JMP) 15.0.0 ver. 3.0.0 (Cary, North Carolina). The continuous data were assessed for normal distribution. Bivariate linear fit and polynomial fit was used to analyze the correlation between the continuous variables, whereas analysis of variance (ANOVA) was used to analyze the correlation of a continuous variable with nominal variables. The whole model test for logistic fit was done using the chi-square test (χ2). The continuous variables were represented as median (Range; Mean ± SD). p<0.05 was considered significant. The survival analysis was done using the Kaplan-Meier method. The log-rank test was used to estimate significance. During analysis, the CT scores were converted to a scale of 0-1 for better interstudy comparison, as internationally, authors used different CTSS scores ranging from 0-15, 0-20 to 0-75.

Data

The data are stored as de-identified participant data which are available on reasonable request to the authors.

## Results

**Figure 1:**
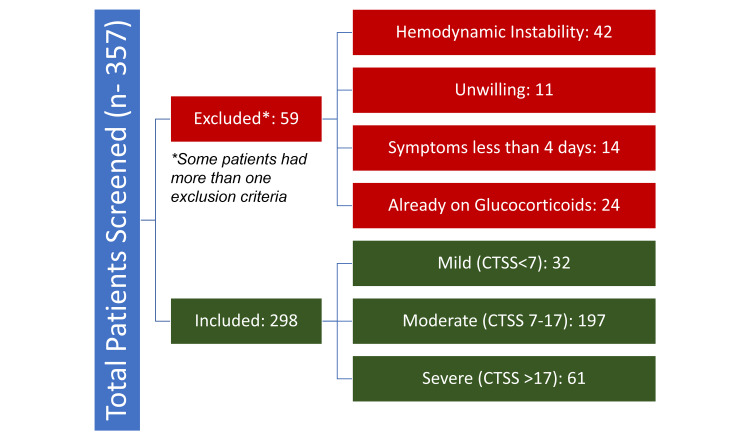
Consort diagram CTSS: computed tomography severity score

**Figure 2:**
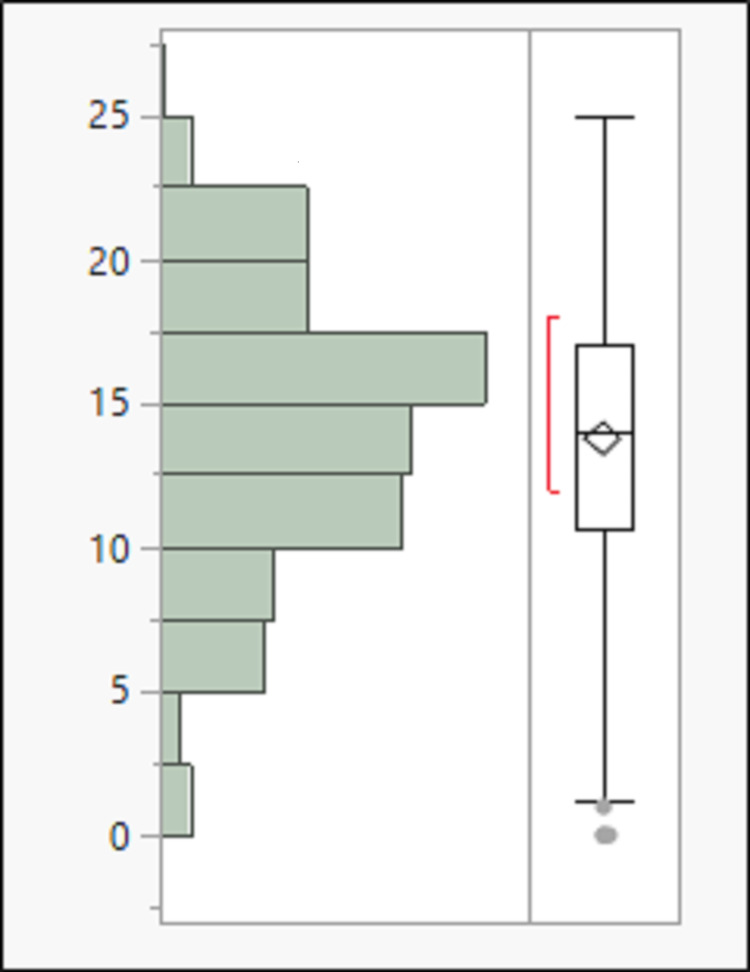
Distribution of CTSS among the study population CTSS: computed tomography severity score

Among the screened population of 357 patients, HRCT was done in 298 patients (study population, 83%) within 24 hours of admission and more than four days of the symptom's onset (Figure 1). The median age of the study population was 59 years (24-95; 58.01±14.89) with a male preponderance (61.41%, n=183). The most common symptoms in the study population were dyspnea, cough, and fever in 73.83% (n-220), 72.15% (n-215), and 58.05% (n-173), respectively. Vaccination by COVISHIELD® (Serum Institute of India Pvt Ltd, New Delhi, India) was carried out in 65 (21.81%) patients, of which 13 (4.36%) had received both doses of vaccine. The median time from the last dose of vaccination to hospital admission was 24 days (0-66; 25.46±13.45). The distribution of the comorbidities and symptoms among the study population is tabulated in Table 1. The distribution of the CT scores among the study population is depicted in Figure 1 and Figure 2. The median duration of hospital stay among the study population was nine days (0-37; 12.2±8.9). A total of 80 patients succumbed to the illness, with 26.85% mortality. The 15 and 30-day survival of the study population was 67.64% and 59.90%.

**Table 1:** Symptoms and comorbidities of the study population

Presence of symptoms/co-morbidities	n	%
Diabetes mellitus	112	37.58
Hypertension	119	39.93
Cerebrovascular disease	11	3.69
Fever	173	58.05%
Cough	215	72.15%
Expectoration	3	1.01%
Dyspnea	220	73.83%
Myalgia	41	13.76%
Diarrhea	11	3.69%
Chest pain	15	5.03%
Headache	23	7.72%
Anosmia	1	0.34%
Sore throat	5	1.68%
Hemoptysis	3	1.01%

**Figure 3:**
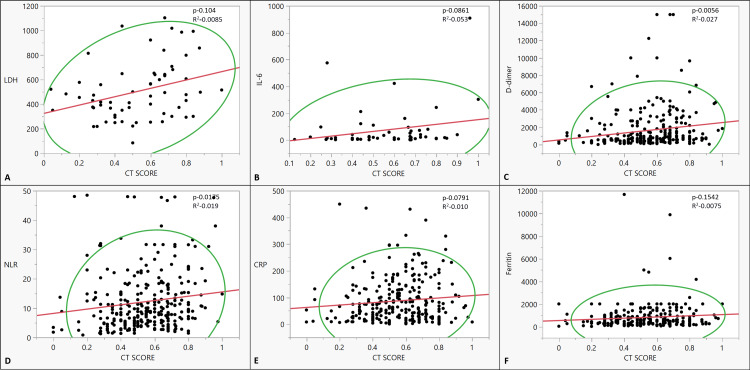
The co-relationship between CT scores and inflammatory markers: (A) LDH (IU/L), (B) IL-6 (ng/dl), (C) D-dimer (ng/ml), (D) NLR, (E) CRP (mg/L), and (F) Serum ferritin (ng/ml) CRP: C-reactive protein; NLR: neutrophilic lymphocytic ratio; IL-6: interleukin-6; LDH: lactate dehydrogenase; CT score: computed tomography score Bivariate linear fit and polynomial fit were used to analyze the correlation and estimate significance.

The CT score of the patients did not correlate with age, gender, and time from vaccination. There was no statistically significant correlation of any of the symptoms of COVID with CT scores. Vaccinated patients had lower CT scores than those who received no vaccination, but the difference was not statistically significant. None of the comorbidities had any correlation with the CT scores (Table 2). On analyzing the relation of various inflammatory markers and CT scores, only LDH, D-dimer, and NLR had a significant correlation (p-0.009, p-0.006, p-0.014, respectively) (Figure 3, Table 3).

**Table 2:** Correlation of the CT score and the symptoms/co-morbidities Severity was defined by pulse oximeter oxygen saturation (SpO2) level at room air of < 90% or respiratory rate > 30 breaths per minute at initial presentation. Hypoxemia was defined as SpO2 < 94%.

Symptoms		n	Median CT score	Mean CT score	p-value
Fever	Present	173	0.56 (0-0.92)	0.55 ±0.19	0.9647
	Absent	125	0.56 (0.44-1)	0.55 ±0.19	
Cough	Present	215	0.55 (0-1)	0.54± 0.19	0.2749
	Absent	83	0.6 (0.05-0.975)	0.57± 0.19	
Expectoration	Present	3	0.675 (0.5-0.72)	0.63± 0.12	0.4403
	Absent	295	0.56 (0-1)	0.55± 0.19	
Dyspnea	Present	220	0.56 (0-1)	0.55± 0.19	0.655
	Absent	78	0.56 (0-0.96)	0.56± 0.19	
Myalgia	Present	41	0.6 (0-0.875)	0.54± 0.2	0.8284
	Absent	257	0.56 (0-1)	0.55± 0.19	
Diarrhea	Present	11	0.64 (0.04-0.875)	0.57± 0.23	0.4866
	Absent	287	0.56 (0-1)	0.55± 0.19	
Chest pain	Present	15	0.56 (0.32-0.875)	0.58± 0.14	0.5966
	Absent	283	0.56 (0-1)	0.55± 0.19	
Headache	Present	23	0.55 (0-0.875)	0.51± 0.24	0.6473
	Absent	275	0.56 (0-1)	0.55± 0.19	
Anosmia	Present	1	0.68	0.68	0.4088
	Absent	297	0.56 (0-1)	0.55± 0.19	
Sore throat	Present	5	0.675 (0.32-0.96)	0.637± 0.23	0.4094
	Absent	293	0.56 (0-1)	0.55± 0.19	
Hemoptysis	Present	3	0.56 (0.55-0.68)	0.6± 0.07	0.6614
	Absent	295	0.56 (0-1)	0.55± 0.19	
Diabetes	Present	112	0.6 (0.2-0.96)	0.56± 0.17	0.4066
	Absent	186	0.55 (0-1)	0.54± 0.20	
Hypertension	Present	119	0.55 (0.05-0.95)	0.55± 0.19	0.5714
	Absent	179	0.56 (0-1)	0.55± 0.19	
Cerebrovascular disease	Present	11	0.625 (0.28-0.8)	0.60± 0.15	0.3221
	Absent	287	0.56 (0-1)	0.55± 0.19	

**Table 3:** Correlation of the CT score and inflammatory markers CRP: C-reactive protein; LDH: lactate dehydrogenase, IL-6: interleukin-6, NLR: neutrophil-lymphocyte ratio

Characteristics			p-value	R^2^
CRP	n	283	0.0791	0.010
	Median (IQR)	63.51(0.56-450)		
	Mean ±SD	86.81±81.7		
LDH	n	65	0.0085	0.104
	Median (IQR)	465(84-1103)		
	Mean ±SD	506.05±231.96		
IL-6	n	56	0.0861	0.053
	Median (IQR)	18.97(1.32-909)		
	Mean ±SD	74.41±155.35		
Serum Ferritin	n	271	0.1542	0.0075
	Median (IQR)	480(11.7-11676)		
	Mean ±SD	818.95±1162.99		
D-dimer	n	280	0.0056	0.027
	Median (IQR)	708(3.5-15000)		
	Mean ±SD	1623.58±2334.67		
NLR	n	284	0.0175	0.019
	Median (IQR)	8.92(0.83-48.48)		
	Mean ±SD	12.20±9.98		

**Figure 4:**
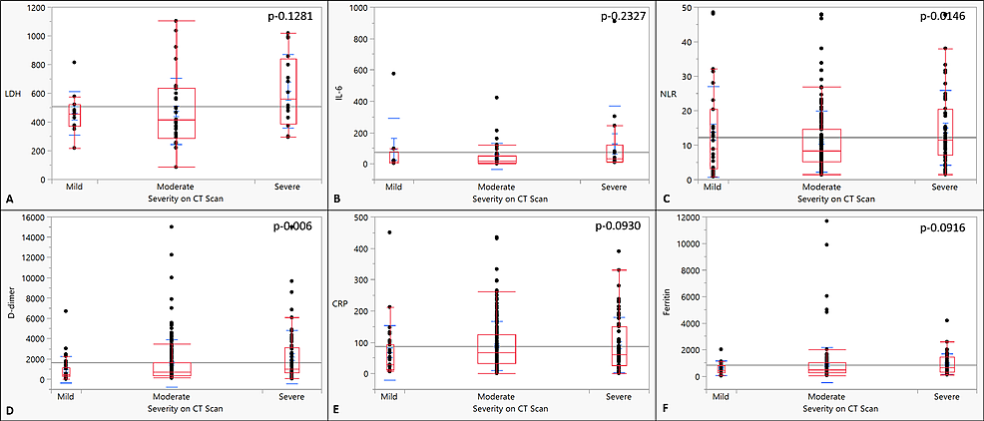
The co-relationship between severity on CT scan and inflammatory markers: (A) LDH (IU/L), (B) IL-6 (ng/dl), (C) NLR, (D) D-dimer (ng/ml), (E) CRP (mg/L), and (F) serum ferritin (ng/ml) CRP: c-reactive protein, NLR: neutrophilic lymphocytic ratio; IL-6: interleukin-6, LDH: lactate dehydrogenase; CT score: computed tomography score Analysis of variance (ANOVA) was used to analyze the correlation and estimate significance.

The mean age was comparable in the three subcategories of CT severity (p-0.29). There was no statistically significant correlation with gender, vaccination, symptoms, and comorbidities between the three CT severity groups (Table 4). Only NLR and D-dimer were significantly different across categories, with severe categories having higher values (p<0.001; 0.015). There was no difference among the groups in other inflammatory markers (Figure 4, Table 5).

**Table 4:** Correlation of CT severity categories with various variables

Characteristics			Mild	Moderate	Severe	p-value
Gender	Male	n	19	120	44	0.8873
		%	6.38	40.27	14.77	
	Female	n	13	77	25	
		%	4.36	25.84	8.39	
Vaccination status	Not vaccinated	n	21	157	55	0.3846
		%	7.05	52.68	18.46	
	First Dose	n	10	31	11	
		%	3.36	10.40	3.69	
	Both Doses	n	1	9	3	
		%	0.34	3.02	1.01	
Diabetes mellitus	Present	n	10	76	26	0.7244
		%	3.36	25.5	8.72	
	Absent	n	22	121	43	
		%	7.38	40.60	62.32	
Hypertension	Present	n	13	76	30	0.7726
		%	4.36	25.5	10.07	
	Absent	n	19	121	39	
		%	6.38	40.60	13.09	
CVD	Present	n	1	6	4	0.6027
		%	0.34	2.01	1.34	
	Absent	n	31	191	65	
		%	10.40	64.09	21.81	
Fever	Present	n	18	114	41	0.9520
		%	6.04	38.26	13.76	
	Absent	n	14	83	28	
		%	4.70	27.85	9.40	
Cough	Present	n	25	142	48	0.6626
		%	8.39	47.65	16.11	
	Absent	n	7	55	21	
		%	2.35	18.46	7.05	
Expectoration	Present	n	0	2	1	0.6814
		%	0	0.67	0.34	
	Absent	n	32	195	68	
		%	10.74	65.44	22.82	
Dyspnea	Present	n	24	147	49	0.8337
		%	8.05	49.33	16.44	
	Absent	n	8	50	20	
		%	2.68	16.78	6.71	
Myalgia	Present	n	6	26	9	0.7057
		%	2.01	8.72	3.02	
	Absent	n	26	171	60	
		%	8.72	57.38	20.13	
Diarrhea	Present	n	1	8	2	0.8890
		%	0.34	2.68	0.67	
	Absent	n	31	189	67	
		%	10.40	63.42	22.48	
Chest pain	Present	n	0	12	3	0.0075
		%	0	4.03	1.01	
	Absent	n	32	185	66	
		%	10.74	62.08	22.15	
Headache	Present	n	4	14	5	0.6048
		%	1.34	4.7	1.68	
	Absent	n	28	183	64	
		%	9.4	61.4	21.48	
Anosmia	Present	n	0	1	0	0.6605
		%	0	0.34	0	
	Absent	n	32	196	69	
		%	10.74	65.77	23.15	
Sore throat	Present	n	0	4	1	0.5366
		%	0	1.34	0.34	
	Absent	n	32	193	68	
		%	10.74	64.77	22.82	
Ageusia	Present	n	0	0	0	
		%	0	0	0	
	Absent	n	32	197	69	
		%	10.74	66.11	23.15	
Hemoptysis	Present	n	0	3	0	0.2867
		%	0	1.01	0	
	Absent	n	32	194	69	
		%	10.74	65.10	23.15	

**Table 5:** Correlation of the CT severity categories and the inflammatory score CRP: c-reactive protein; LDH: lactate dehydrogenase; IL-6: interleukin-6; NLR: neutrophil-lymphocyte ratio

Characteristics		Mild	Moderate	Severe	p-value
CRP	n	31	190	62	0.0930
	Median (IQR)	29(6.2-450)	66.6(0.6-434.9)	60.4(0.56-390)	
	Mean ±SD	66.52±86.98	78.33±5.68	88.83±11.28	
LDH	n	11	38	16	0.1281
	Median (IQR)	454(217-814)	413(84-1103)	560.5(293-1018)	
	Mean ±SD	460.36±151.89	473.66±231.09	614.36±256.41	
IL-6	n	8	34	14	0.2327
	Median (IQR)	15.75(3.7-575)	17.62(1.32-422)	31.45(9.61-909)	
	Mean ±SD	93.33±196.95	47.85±82.53	128.1±242.39	
Serum ferritin	n	30	182	59	0.0916
	Median (IQR)	396.5(11.7-2000)	464.5(37.93-11676)	610(89.7-4171)	
	Mean ±SD	588.87±550.84	832.05±1329.09	895.53±773.37	
D-dimer	n	31	188	61	0.0006
	Median (IQR)	434(3.5-6694)	682.92(100-15000)	1004(33.7-15000)	
	Mean ±SD	932.56±1307.80	1556.20±2330.89	2182.42±2641.77	
NLR	n	31	190	63	0.0146
	Median (IQR)	8.92(0.83-48.48)	8.40 (1.36-48)	11.4 (1.46-47.9)	
	Mean ±SD	13.80±13.15	11.00±8.87	15.01±10.84	

**Figure 5:**
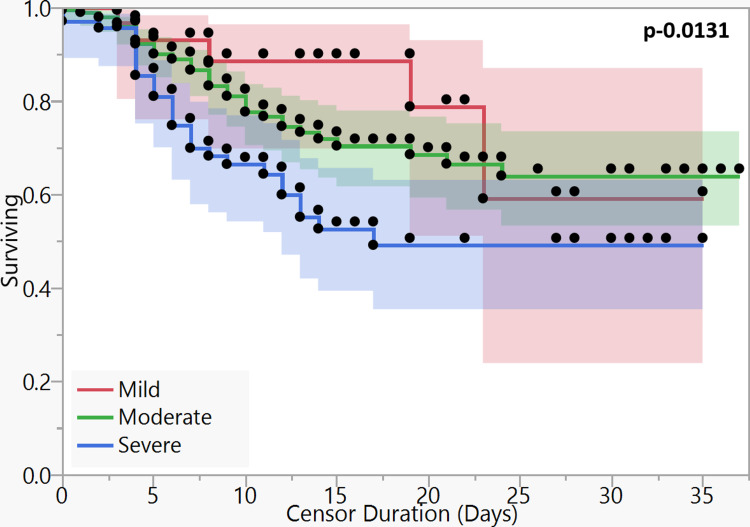
Kaplan-Meier curves for survival based on severity on CT scan ꭕ2 – 8.667, dF – 2, p-0.0131 The log-rank test was applied to estimate significance.

On survival analysis of different CTSS categories, the patients with severe CT scores had inferior survival (p-0.013) (Figure 5). The 15-day overall survival percentages (15-d OS) of mild, moderate, and severe CT categories were 88.62%, 70.39%, and 52.62%, respectively, while 30d-OS of three categories were 59.08%, 63.96%, and 49.12%, respectively. The median OS of the severe category was 16 days.

## Discussion

In our cohort of 298 hospitalized, hypoxemic COVID-19 patients, CT thorax showed a good correlation with 15-day and 30-day survival. The CT findings did not show any meaningful correlation with other patient demographic or laboratory parameters.

CT thorax is a good predictor of the severity of the disease [17-19]. The use of a severity score based on CT findings provides objectivity to the CT interpretation and allows correlation with other factors related to the disease process and outcomes. There is no consensus on the CT severity scoring system. A visual score used in our study has previously been shown to correlate well with disease severity. It has acceptable inter-observer variability with an intragroup correlation coefficient greater than 0.9, suggesting wider clinical applicability because of decreased subjectivity [11,16]. Similar findings have been obtained when automated or semi-automated software was used to analyze CT acquisition data. Compared with non-severe disease, patients with severe disease have a higher total score and higher individual scores of ground-glass opacities (GGO) consolidation. Also, the severe disease has a higher percentage of consolidation but a lower percentage of GGO relative to total lesion volume [7,20]. It has been shown that there is a significant difference in CT scores of scans taken in the first four days from symptom onset and those taken after Day 5, beyond which the scores remain relatively stable in most cases, with the peak around day 10 [2,15,21]. In all our cases, a CT scan was done after a minimum of four days from symptom onset.

Our study population was different from most previous studies because all our patients had baseline hypoxia, were admitted as severe disease, and were steroid-naïve till CT thorax was conducted and blood samples were drawn. High-resolution CT thorax showed no association with any of the demographic and patient characteristics in our study. A few studies in the past have shown higher CT scores in the male population [17,22]. This finding has not been replicated in other studies [23]. Also, the denominator in these studies was remarkably different from our study as these included a large number of mild to moderate cases.

In our study, only NLR, D-dimer, and LDH had a significant correlation with the CT score. Also, only NLR and D-dimer had significant differences across CT severity score categories. A surprising finding in our study was that the CT score did not show a correlation with CRP values. This is in contrast with several previously published studies [5]. Most of these studies, however, involved patients of all severity categories. We exclusively studied patients who were admitted due to severe disease and hypoxemia. Liu et al. studied various predictors of outcome in their hospitalized COVID-19 patients. After multivariate analysis, only CT score and lymphocyte count at admission, and not CRP, were found to independently influence the outcome [24]. A study of 93 admitted patients at Wuhan showed that the CT score correlated positively with LDH, D-dimer, and erythrocyte sedimentation rate (ESR) but not CRP and correlated negatively with lymphocyte count, as in our study [25]. It has also been shown that in severe disease, CRP levels rise steeply in the first week of illness, even before the CT manifestations appear, and after that fall rapidly [10]. Zhang et al. showed that CRP and other laboratory parameters correlated with CT findings only in the initial stages, whereas there was no correlation of CT score with laboratory parameters thereafter [23]. CRP, thus, may be a good predictor of disease severity in the initial period, but once the disease becomes severe, the CT score may correlate better with outcomes than CRP and other inflammatory markers.

Most studies have utilized the visual scoring system used in this study, where each lobe is scored separately (from 0 to 5) and the maximum score is 25 [5,8-9,15,17,26]. Li et al. projected that a cutoff of 7 predicted severe or critical disease with a sensitivity and specificity of 80% and 82.8%, respectively [26]. Francone et al. used the Kaplan-Meir analysis and projected that a CTSS equal to or more than 18 significantly increased the risk of mortality [5]. Mahdjoub reported in their study that the optimal CTSS cutoff for poor five-day outcomes was 13 [8]. Similarly, Lieveld et al. estimated that CTSS cutoffs of 10, 15, and 17 predicted hospital admission, ICU admission, and mortality, respectively, with a specificity of more than 90% [9]. Some authors have utilized a slightly different scoring system, wherein the lungs have been divided into six zones and each zone could be scored from 0 to 4 and 24 was the maximum total score possible [12,18,21,25]. Using this system, Khosravi et al. estimated that a total score of greater than eight correlated with ICU admission, intubation, and mortality [18]. Few authors have scored each lobe from 0 to 4 and thus the total score ranged from 0 to 20 [16,24,27]. Another scoring method has been utilized by some groups, where each of 20 bronchopulmonary segments have been scored from 0 to 2 and thus the total maximum score is 40 [11]. There has been no comparison between the various scoring systems, and each of these has been shown to have an excellent inter-reader agreement [28].

Similarly, severity groups based on CTSS have varied in the literature. We divided the groups as CTSS less than 7, 7-17, and greater than 17. This categorization has been shown to have a significant correlation with lymphopenia, CRP, ferritin, D-dimer, length of hospitalization, oxygen requirement, and ICU admission [6]. Other cutoffs for CTSS categorization have been used, like 1-5, 6-14, and 15-25 for the mild, moderate, and severe CTSS categories, respectively [29]. Rutkowska et al. arbitrarily categorized CTSS as 1-5, 6-10, 11-15, and 16-25 as mild, moderate, severe, and critical, respectively. They showed that the critical CTSS category had more lymphopenia and higher IL-6 than non-critical cases [30]. No categorization based on CTSS has been validated to be superior to others to date [28].

In this study, CT score on admission was a good predictor of outcome in terms of survival at Day 15 and Day 30. A recent review of published literature found that CT thorax at presentation has a significantly higher severity score in non-survivors compared to that in survivors, with a higher proportion of consolidations and central opacities in non-survivors [3]. Francone et al. studied the correlation of CT thorax with mortality, patient characteristics, and laboratory parameters during the initial months of the pandemic. Most patients in this study had mild disease. CT thorax showed a good correlation with mortality, CRP, and D-dimer. However, after multivariate analysis for predictors for death, only CT and age remained significant and not CRP or D-dimer [5]. Khosravi et al. recently showed that a CT score of more than eight at admission predicts a three-fold increase in the probability of ICU admission, intubation, and mortality [18].

The major strength of our study comes from the fact that we restricted our analysis specifically to severe COVID-19 patients who had hypoxia on admission. This is important because patients in this category have the worst outcomes and prognostication with CT thorax at admission may have profound implications. It is therefore noteworthy that the CT thorax maintains its prognostic prowess even among patients in the severe category. Another major strength in our study was the fact that all included patients were steroid naïve. Thus, any blunting of the inflammation due to the effect of steroids has been ruled out.

There are inherent limitations in the study due to its retrospective design. Being a single-center study, the possibility of demographic population characteristics and hospital protocols on the outcomes and study results cannot be ruled out. Interobserver variability in the interpretation of CT scores was not checked, although an excellent interobserver agreement intragroup has been described in previous studies [11,16]. All patients in our study underwent a CT scan within 24 hours of admission. However, the impact on results due to variability in duration between onset of symptoms and CT scan was not accounted for.

## Conclusions

In conclusion, among hospitalized COVID-19 patients with hypoxia on admission, CT thorax severity scores correlate well with survival at 15 and 30 days. The CTSS also has a significant, although weak, correlation with NLR, D-dimer, and ferritin levels but not with CRP and IL-6. Multicenter longitudinal studies are required to confirm if CT thorax may be a better predictor of outcomes than blood inflammatory markers in hypoxic hospitalized COVID-19 patients.
